# Glycyrrhizin improves p75NTR-associated sciatic nerve regeneration in a BALB/c mouse model

**DOI:** 10.3892/etm.2014.1546

**Published:** 2014-02-14

**Authors:** YU-XI JIA, JIN-RAN LI, CUI-YING MAO, WEI-TIAN YIN, RI-HUA JIANG

**Affiliations:** 1 Department of Orthopaedics, China-Japan Union Hospital of Jilin University, Changchun, Jilin 130033, P.R. China; 2 Jilin University Bethune School of Medical Sciences, Changchun, Jilin 130021, P.R. China; 3 Department of Dermatology, China-Japan Union Hospital of Jilin University, Changchun, Jilin 130033, P.R. China

**Keywords:** glycyrrhizin, peripheral nerve injury, p75 neurotrophin receptor, sciatic nerve, regeneration

## Abstract

Glycyrrhizin has a role in immune regulation in the central nervous system, but its impact on sciatic nerve injury had not previously been reported. In this study, a BALB/c mouse model of sciatic nerve injury was used to explore the role of glycyrrhizin in sciatic nerve repair and its underlying mechanism. Glycyrrhizin with intragastric gavage of 10 and 20 mg/kg weight per day (mid- and high-dose, respectively) inhibited p75 neurotrophin receptor (p75NTR) expression at the protein and mRNA levels versus the 5 mg/kg (low-dose) group and control (0.9% NaCl solution) at one, two, four and eight weeks following sciatic nerve injury, and simultaneously improved the action potential amplitude and motor nerve conductive velocity. Combined Marsland, Glees and Erikson’s silver stain and Luxol fast blue staining results indicated that high- and mid-dose glycyrrhizin promoted improved sciatic nerve myelination compared with the low-dose or control groups eight weeks after injury. Immunofluorescence staining demonstrated that glycyrrhizin had an inhibitory effect to a certain degree on local hypertrophic scar and inflammatory responses in the mouse model. In conclusion, glycyrrhizin can promote sciatic nerve regeneration and functional repair, in which doses of 10 and 20 mg/kg per day are more effective than lower doses, and such regeneration is associated with the downregulation of p75NTR.

## Introduction

Glycyrrhizin, a naturally occurring licorice flavonoid glycoside extracted from licorice (*Glycyrrhiza uralensis* Fisch) root has been reported to have multiple functions (1–3); it can scavenge free radicals, have anti-bacterial, anti-inflammatory, antiviral and anti-ulcer effects and protect cytochrome enzymes as an antioxidant (1–6). Studies have shown that glycyrrhizin can regulate the secretion of various cytokines and the functioning of immunomodulatory effects in the central nervous system (6,7). Glycyrrhizin can also improve intracerebral ischemic injury (6,8,9). During 20 years of anti-inflammatory application for the treatment of liver diseases, glycyrrhizin has not generated any toxicity or side-effects (10–12). However, the effects of glycyrrhizin on functional recovery following peripheral nerve injury are not well understood.

The p75 neurotrophin receptor (p75NTR), one of the nerve signaling proteins, plays a significant role in neuronal survival, apoptosis and axonal growth in the peripheral and central nervous systems. p75NTR, as an apoptosis-inducing receptor, has been demonstrated to induce cell death in developing peripheral sympathetic neurons and early retinal neurons (13–17). Küst *et al* found that p75NTR is also associated with the inflammatory response of autoimmune encephalomyelitis in C57BL/6 mice (18). The present study explored the reparative effects of glycyrrhizin on sciatic nerve injury and the p75NTR-associated immune inflammatory response in a BALB/c mouse model.

## Materials and methods

### Reagents and animals

**Figure 1:**
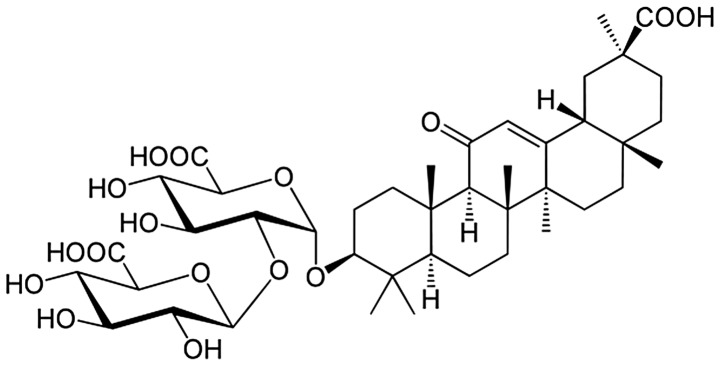
Structural formula of glycyrrhizin.

Glycyrrhizin (IUPAC name, [3β,18α]-30-hydroxy-11,30-dioxoolean-12-en-3-yl 2-O-β-D-glucopyranuronosyl-β-D-glucopyranosiduronic acid; CAS number, 1405-86-3; molecular formula, C_42_H_62_O_16_; molecular weight, 822.93 Da; purity, 98%) was purchased from Xi’an Tianxingjian Natural Bio-products Group (Xi’an, Shaanxi, China), and its structure is shown in Fig. 1. In total, 160 healthy adult male BALB/c mice (weighing 25±2 g) were housed and fed in Jilin University School of Medicine [Experimental Animal Center, Changchun, China; License No. SCXK (Kyrgyzstan) 2007-0001], with a normal diet, access to water *ad libitum* and room temperature conditions. The animal experiments were approved by Jilin University Ethics Committee.

### Modeling and treatment

The sciatic nerve injury model was created as previously described (19,20), with certain modifications. Briefly, all mice underwent intraperitoneal injection of 3% ketamine using 100 mg/kg weight for anesthesia. The two cm-longitudinal incisions were aseptically made on the unilateral rear thigh. The sciatic nerve cords were completely interrupted at 0.5 cm below the ischial tuberosity and immediately underwent microsurgical anastomosis using 11-0 microsutures (Sharpoint™ MicroSutures, Angiotech Pharmaceuticals, Inc., Vancouver, BC, Canada). Finally, the muscle and skin were sutured. Mice were randomized into four groups of 40 mice each and treated with different concentrations of glycyrrhizin for four different time periods. Glycyrrhizin was dissolved in 0.9% NaCl solution and administered intragastrically, daily for 14 continuous days. The final high-, mid- and low-doses were 20, 10 and 5 g/kg weight/day, respectively. An NaCl solution (0.9%) of an equivalent volume served as the control. Mice were sacrificed one, two, four and eight weeks after injury.

### Electroneurophysiological testing

Affected sciatic nerves underwent electroneurophysiological testing using an electromyograph and evoked potential machine (Keypoint^®^, Medtronic A/C Inc., Skovlunde, Denmark) prior to the mice being sacrificed, as previously described (20), with certain modifications. Briefly, the sciatic nerves were exposed at 24°C, then pin electrodes were pierced into the musculi soleus for wave recording (M point) and grounding electrodes were placed on the tails. A single current stimulus (10 mA) was employed to stimulate proximal anastomotic ischial tuberosity (P point) and the distal sciatic nerve bifurcation point (D point), respectively, using a parallel stimulating electrode with a fixed space of 3.3 mm between two tips. Motor nerve conductive velocity (MNCV) equaled the distance between the P and D points divided by the difference value of potential latency.

### Preparation of spinal cord specimens

The affected spinal cord segments, L4–L6, connected to injured sciatic nerves, were dissected, dissociated and removed as previously described (19,20). Tissues were stored in liquid nitrogen until use in quantitative polymerase chain reaction (qPCR) and western blotting analysis. Alternatively, tissues were soaked in 10% neutral buffered formalin for fixation for 72 h, then dehydrated by gradient alcohols and embedded in paraffin for use.

### Western blotting and qPCR analysis

Western blotting procedures were routinely performed, as previously described (20,21), but the goat anti-rat p75NTR polyclonal antibodies (Santa Cruz Biotechnology, Inc., Dallas, TX, USA) were diluted 100 times with phosphate-buffered saline containing 1% bovine serum albumin (Beyotime Inc., Nanjing, China). In the qPCR experiments, the total RNA was extracted from the tissues using the TRIzol (Invitrogen, Carlsbad, CA, USA) reagent method. cDNA was synthesized by reverse transcription using total RNA templates. p75NTR mRNA was detected by qPCR using cDNA templates. Reduced glyceraldehyde-phosphate dehydrogenase (GAPDH) served as a housekeeping gene in each reaction system. The relative p75NTR/GAPDH mRNA levels were determined. The reaction conditions were 35 cycles at 95°C for 30 sec, 57°C for 60 sec and 72°C for 60 sec. p75NTR and GAPDH primers were designed based on the NCBI Genbank sequences using Beacon Designer 7 software (Premier Biosoft Inc., Palo Alto, CA, USA) and synthesized by Sangon Bioengineering, Inc. (Shanghai, China). The p75NTR primer sequences were: Sense, 5′-AGTGGCGGATATGGTGAC-3′; antisense, 5′-GAGCAATAGACAGGAATGAGG-3′; and probe, 5′-TCC TGACTCCGTTGCTGCTCCCGA-3′. The GAPDH primer sequences were: Sense, 5′-AATGTGTCCGTCGTGGAT CTG-3′; antisense, 5′-CAACCTGGTCCTCAGTGTAGC-3′; and probe, 5′-CGTGCCGCCTGGAGAAACCTGCC-3′.

### Histological examination

Paraffin-embedded tissues were sectioned into transverse slices of 3 μm in thickness. Slices were stained using combined Marsland, Glees and Erikson’s silver stain (Marsland’s) and Luxol fast blue (LFB) staining methods, as previously described (21,22). Nerve fibers were stained black and myelin was stained blue (23). In each slice, five microscopic fields of ×400 magnification were randomly selected and their images captured. The diameter and number of myelinated fibers was calculated using image analysis software Image-Pro^®^ Plus 6.0 (Media Cybernetics Inc., Silver Spring, MD, USA).

### Immunofluorescence staining

Paraffin-embedded tissues were sectioned into slices of 3 μm in thickness. Indirect immunofluorescence staining was routinely performed to detect expression in astrocytes. Glial fibrillary acidic protein (GFAP) was stained blue and neuron-specific enolase (NSE) was stained green. Goat anti-rat GFAP and NSE monoclonal antibodies (Santa Cruz Biotechnology, Inc.) were diluted 1,000 times. The fluorescence intensity of five random fields was measured using a confocal laser scanning microscope (FV1200CLSM; Olympus Inc., Beijing, China). The excitation wavelengths were 488 nm and 561 nm for blue and green fluorescence, respectively.

### Statistical analysis

Data are presented as the mean ± standard deviation. The SPSS 17.0 statistical package (SPSS, Inc., Chicago, IL, USA) was used for the statistical analysis. Student’s t-test was used for comparisons between groups. P<0.05 was used to indicate a statistically significant difference.

## Results

### Electrophysiological testing

The functional recovery of the sciatic nerves was determined by the action potential amplitude and MNCV (Table I). The amplitude and MNCV of the high- and mid-dose groups at one, two, four and eight weeks after injury were significantly higher than for the low-dose and control groups (P<0.05). There was no statistically significant difference between the high- and mid-dose groups or between the low-dose and control groups. Administration of glycyrrhizin at 10 and 20 mg/kg/day accelerated the functional recovery of the injured sciatic nerves in the mice.

### Western blotting and qPCR analysis

**Figure 2:**
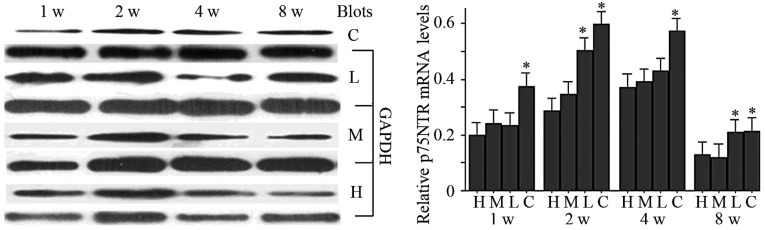
p75NTR protein blots and relative p75NTR/GAPDH mRNA levels in high- (H), mid- (M) and low-dose (L) and control (C) groups at the time-points of one, two, four and eight weeks after injury. ^*^P<0.05 vs. high- and mid-dose groups (Student’s t-test). Values are presented as the mean ± standard deviation, n=5. p75NTR, p75 neurotrophin receptor; GAPDH, glyceraldehyde-phosphate dehydrogenase.

The western blotting analysis of the results is shown in Fig. 2, and the blot grayscale ratio in Table II. Levels of p75NTR protein in each group increased markedly between one and two weeks after injury, and then decreased markedly between two and eight weeks. At each time-point, each glycyrrhizin treatment group had significantly reduced p75NTR protein expression versus the control group (P<0.05 for each). Meanwhile, the high- and mid-dose groups at one and four weeks showed lower levels of p75NTR protein versus the low-dose groups (P<0.05 for each), and the high-dose group expressed significantly reduced levels versus the low-dose group at two weeks (P<0.05). The qPCR analysis of the results is shown in Fig. 2. Overall, the levels of p75NTR mRNA in each group increased following injury and then decreased. The levels of p75NTR mRNA in the low-dose and control groups increased between one and four weeks and then decreased between four and eight weeks. While a decrease in p75NTR mRNA expression in the high- and mid-dose groups occurred early between two and eight weeks, p75NTR mRNA expression in the high- and mid-dose groups was significantly lower versus the control (P<0.05) at each time-point. Application of glycyrrhizin, particularly using doses of 20 and 10 mg/kg/day, markedly inhibited the expression of p75NTR at the protein and mRNA levels.

### Histological staining

**Figure 3:**
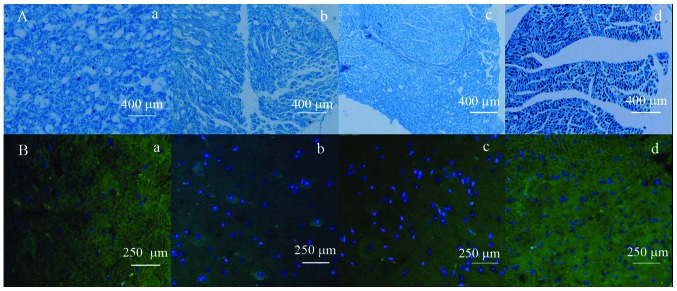
Stained transverse slices eight weeks after injury. (A) Combined Marsland, Glees and Erikson’s silver stain and Laxol fast blue staining (light microscopy, ×400). Nerve fibers are stained black and myelin is stained blue. (B) Indirect immunoflurescence staining of astrocytes detected by confocal laser scanning microscopy. Glial fibrillary acidic protein (GFAP) staining appears as blue dots and neuron-specific enolase (NSE) staining appears as an abundance of green in the cytoplasm. (a) High-dose group, (b) mid-dose group, (c) low-dose group, (d) control.

Nerve fibers and myelin were dually stained using combined Marsland’s and LFB staining methods to detect nerve regeneration and myelinization at eight weeks after injury. Nerve fibers were stained black and myelin was stained blue (Fig. 3A). Myelin sheaths in the high- (Fig. 3Aa) and mid-dose groups (Fig. 3Ab) were regularly shaped, having uniform thickness and possessing clear borders or little fibrous hyperplasia. Myelin sheaths in the low-dose group were of irregular shape and thickness, and possessed clear borders and visible fibrous hyperplasia (Fig. 3Ac). Myelin sheaths in the control group were extremely irregularly shaped with marked fibrous hyperplasia (Fig. 3Ad). Based on these dual stained images, the number of myelinated fibers in five random microscopic fields and their average diameters were analyzed using image analysis software Image-Pro^®^ Plus 6.0 (Table III). The number and diameter of myelinated fibers in the high- and mid-dose groups at each time-point were significantly higher than in the low-dose and control groups, respectively (P<0.05). There was no statistically significant difference between the high- and mid-dose groups or between the low-dose and control groups. Using 10 and 20 mg/kg doses of glycyrrhizin per day accelerated nerve regeneration.

### Immunofluorescence staining

The expression of GFAP and NSE in astrocytes was detected by indirect immunofluorescence staining at eight weeks after injury. GFAP staining appeared as blue dots and NSE stains appeared as an abundance of green in the cytoplasm (Fig. 3B). Overall, GFAP and NSE were expressed in each of the four groups. The high-dose group (Fig. 3Ba) was less expression of GFAP, as indicated by fewer blue dots and more expression of NSE, as indicated by more green dots in cytoplasm, than the mid-dose (Fig. 3Bb) and low-dose (Fig. 3Bc) groups. The mid-dose and low-dose groups were less expression of GFAP and more NSE in the cytoplasm than the controls (Fig. 3Bd). In the control group, certain specimens were observed to form glial scars. Fluorescence densities in each group are shown in Table III. Each glycyrrhizin treatment group had less blue fluorescence and more green fluorescence than the controls (P<0.05 for each). The high-dose group had less blue fluorescence and more green fluorescence than the low-dose and control groups (P<0.05 for each). The application of glycyrrhizin increased NSE and reduced GFAP expression in the astrocytes in order to improve sciatic nerve repair.

## Discussion

In the present study, 10 and 20 mg/kg doses of glycyrrhizin were shown to promote the functional recovery of sciatic nerves. This was verified by measuring the potential amplitude and MNCV in mice. Dual staining of the affected spinal cord segments, L4–L6, connected to injured sciatic nerves, was performed to examine the structural evidence that 10 and 20 mg/kg doses of glycyrrhizin can elevate the number and diameter of myelinated fibers, accelerating nerve regeneration. Subsequently, the GFAP- and NSE-marked fluorescence in astrocytes was employed to assess the nerve damage-associated molecules. Upregulation of GFAP in astrocytes partially marks the formation of glial scars, showing that astrocytes interact with fibrous tissues to reconstruct the glial layers around the central injury core (24,25). NSE, a homodimer, is a biomarker for mature neurons and neuroendocrine cells (26). High-dose glycyrrhizin results in increased NSE and reduced GFAP expression in astrocytes, indicating sciatic nerve repair.

Nerve repair and regeneration following injury involves a complex biological process, including an inflammatory response, an adhesion reaction, a synergistic effect of the extracellular matrix, the regulatory function of neurotrophic factors on the regeneration, synthesis and release of a neurotransmitter, the formation and extension of a growth cone and final neuronal regeneration (9,14). Following nerve injury, the local excessive immune response leads to expression of inflammatory response genes, forming a local glial scar. This scar can directly affect the recovery of nerve conduction. Reducing the scar can provide a favorable external environment for the repair and regeneration of nerves.

In the present study, glycyrrhizin, particularly at doses of 10 and 20 mg/kg, markedly inhibited the expression of p75NTR at the protein and mRNA levels throughout the eight weeks of the experiment. This downregulation of p75NTR synchronizes the functional recovery of the sciatic nerve, structural myelination and neuronal regeneration and the improvement of molecular markers. Considering p75NTR is an apoptosis-inducing receptor and is associated with the inflammatory response of autoimmune encephalomyelitis (13–18), the downregulation of p75NTR may likely result in the reduction of the local inflammatory response, lessening the glial scar and recovering nerve conduction function. Simultaneously, downregulation of p75NTR may attenuate the apoptosis-inducing receptor function of p75NTR, promoting neuronal regeneration and the expression of marker molecules. Therefore, sciatic nerve regeneration is associated with the downregulation of p75NTR.

In conclusion, 10 and 20 mg/kg doses of glycyrrhizin per day are effective in promoting sciatic nerve regeneration and functional repair, and such regeneration or repair is associated with the downregulation of p75NTR.

## Figures and Tables

**Table I:** Neuroelectrophysiological potential amplitude (mV) and MNCV (m/sec) of each dose group at each time-point following injury (n=10).

Glycyrrhizin, mg/kg/day	Action potential amplitude, mV				MNCV, m/sec			
	
	1 week	2 weeks	4 weeks	8 weeks	1 week	2 weeks	4 weeks	8 weeks
20 (High)	2.32±0.21^a^	4.64±0.17^a^	26.13±0.16^a^	26.44±0.68^a^	20.8±0.31^a^	41.9±0.59^a^	64.8±1.73^a^	68.8±1.34^a^
10 (Mid)	2.29±0.16^a^	4.39±0.19^a^	21.87±0.26^a^	23.11±0.33^a^	19.6±0.38^a^	35.5±0.72^a^	55.7±1.33^a^	60.4±0.61^a^
5 (Low)	1.40±0.13	2.66±0.07	17.56±0.32	19.76±0.36	13.0±0.19	30.6±0.84	52.0±0.66	55.6±0.43
0 (Control)	1.31±0.10	2.19±0.15	13.29±0.06	15.77±0.68	10.4±0.17	27.6±0.48	49.1±0.34	46.9±2.17

a P<0.05 vs. low-dose and control groups (Student’s t-test).

Values are presented as the mean ± standard deviation. MNCV, motor conductive nerve velocity.

**Table II:** Relative grayscale of p75NTR/GAPDH blots (n=5) of each dose group at each time-point following injury.

	Relative grayscale			
	
Glycyrrhizin, mg/kg/day	1 week	2 weeks	4 weeks	8 weeks
20 (High)	0.512±0.020^a^	1.088±0.022^a^	0.697±0.032^a^	0.233±0.025^b^
10 (Mid)	0.599±0.026^a^	1.207±0.022^b^	0.758±0.033^a^	0.232±0.019^b^
5 (Low)	0.874±0.021^b^	1.333±0.022^b^	1.111±0.020^b^	0.288±0.030^b^
0 (Control)	1.311±0.035	1.735±0.24	1.432±0.013	0.523±0.004

a P<0.05 vs. low-dose and control groups;

b P<0.05 vs. control group (Student’s t-test).

Values are presented as the mean ± standard deviation. p75NTR, p75 neurotrophin receptor; GAPDH, glyceraldehyde-phosphate dehydrogenase.

**Table III:** Myelinated fiber status and immunofluorescence intensity eight weeks after injury (n=5).

Glycyrrhizin, mg/kg/day	Myelinated fiber count, n/mm^2^	Myelinated fiber diameter, μm	GFAP fluorescence intensity (a.u.)	NSE fluorescence intensity (a.u.)
20 (High)	76±3^a^	2.46±0.30^a^	133.21±24.18^a^	466.26±23.12^a^
10 (Middle)	70±1^a^	2.30±0.17^a^	222.88±21.32^b^	298.48±32.77^b^
5 (Low)	55±3	1.96±0.31	246.39±15.88^b^	288.02±30.15^b^
0 (Control)	49±1	1.56±0.25	299.74±17.31	178.71±15.13

a P<0.05 vs. low-dose and control groups;

b P<0.05 vs. control group (Student’s t-test).

Values are presented as the mean ± standard deviation. GFAP, glial fibrillary acidic protein; NSE, neuron-specific enolase.
